# Modeling Zero-Dose Children in Ethiopia: A Machine Learning Perspective on Model Performance and Predictor Variables

**DOI:** 10.2196/76712

**Published:** 2026-02-02

**Authors:** Berhanu Fikadie Endehabtu, Kassahun Alemu, Shegaw Anagaw Mengiste, Meseret Zelalem, Monika Knudsen Gullslett, Binyam Tilahun

**Affiliations:** 1 Department of Health Informatics, Institute of Public Health, College of Medicine and Health Sciences, University of Gondar, Gondar, 196, Ethiopia, 251 921013129; 2 Center for Digital Health and Implementation Science, University of Gondar, Gondar, Ethiopia; 3 Department of Epidemiology and Biostatistics, Institute of Public Health, College of Medicine and Health Sciences, University of Gondar, Gondar, Ethiopia; 4 School of Business, University of South-Eastern Norway, Drammen, Norway; 5 Department of Pediatrics and Child Health, University of Gondar, Gondar, Ethiopia; 6 Norwegian Centre for E-health Research, University Hospital of North Norway, Tromsø, Norway

**Keywords:** modeling, zero dose, children, machine learning, Ethiopia

## Abstract

**Background:**

Despite progress in childhood vaccination, many children in low- and middle-income countries, including Ethiopia, remain unvaccinated, presenting a significant public health challenge. The Immunization Agenda 2030 (IA2030) seeks to halve the number of unvaccinated children by identifying at-risk populations, but effective strategies are limited. This study leverages machine learning (ML) to identify Ethiopian children aged 12-35 months who are at higher risk of being zero dose (ZD). By analyzing demographic, socioeconomic, and health care access data, the study developed predictive models using different algorithms. The findings aim to inform targeted interventions, ultimately improving vaccination coverage and health outcomes.

**Objective:**

This study aimed to develop an ML model to predict ZD children and to identify the most influential predictors of ZD in Ethiopia.

**Methods:**

We examined how well the predictive algorithms can characterize a child at risk of being ZD based on predictor variables sourced from the recent National Immunization Evaluation Survey data. We applied supervised ML algorithms with the survey datasets, which included 13,666 children aged 12-35 months. Model performance was assessed using accuracy, area under the curve, precision, recall, and *F*_1_-score. We applied Shapley Additive analysis to identify the most important predictors.

**Results:**

The Light Gradient Boosting Machine (LGBM), Random Forest, Extreme Gradient Boosting (XGBoost), and AdaBoost classifiers effectively identified most ZD children as being at high risk. Among these, LGBM demonstrated the best performance, achieving an accuracy of 93%, an area under the curve of 97%, a precision of 94%, and a recall of 91%. The most significant features impacting the model included poor perception of vaccination benefits, lack of antenatal care utilization, distance from immunization services, and absence of maternal tetanus toxoid vaccinations.

**Conclusions:**

The developed ML models effectively predict children at risk of being ZD, with the LGBM model showing the best performance. This model can guide targeted interventions to reduce ZD prevalence and address vaccination inequities. Key predictors include access to immunization sites, maternal health service utilization, and perceptions of immunization benefits. By focusing on these vulnerable groups, public health efforts can tackle disparities in vaccination coverage. Enhancing maternal care, raising caregiver awareness, and improving immunization access through outreach can significantly reduce the number of ZD children.

## Introduction

Child immunization is a cornerstone of public health, essential for safeguarding against life-threatening diseases and promoting the health of future generations [1]. Globally, significant advancements have been made in immunization programs, resulting in higher coverage rates [2] and a corresponding decline in vaccine-preventable disease [3]. However, as of 2023, approximately 14.5 million children worldwide didn’t receive the first dose of diphtheria, tetanus, and pertussis (DTP1) containing vaccines [4], a widely used indicator of access to immunization services [5]. This high number of zero-dose (ZD) children continues to be a pressing issue, intensifying health inequalities and heightening the likelihood of vaccine-preventable disease outbreaks [6]. These ZD children remain at high risk, creating considerable hurdles for public health efforts [7-9].

In a substantial portion of these ZD children, about 60% are concentrated in 10 low- and middle-income countries, including Ethiopia. Despite notable achievements in improving immunization coverage in Ethiopia, the country ranks third globally for ZD children, following Nigeria and India, accounting for 6% of the world’s total [4].

Addressing the issue of children at risk of becoming ZD has emerged as a priority on both national and global agendas [10]. The Immunization Agenda 2030 (IA2030), endorsed by the World Health Assembly in November 2020, aims to reduce the number of ZD children by ensuring that every child is reached by 2030 [5]. However, effectively identifying and reaching these at-risk children poses significant operational challenges, and little is known about what strategies perform best.

Research in Ethiopia has identified various predictors of low immunization uptake, including low education levels and low wealth index [11-13], rural residence [12,14], limited access to health services [15,16], lack of antenatal care (ANC) and postnatal care (PNC) [13,15-18], home deliveries [13-16], absence of maternal tetanus toxoid (TT) vaccination [12,19], and poor caregiver knowledge [16]. However, there is a lack of evidence regarding how well these factors predict ZD status specifically and which factors are most relevant for optimal prediction.

Recent advancements in data science, coupled with available routine immunization data, present new opportunities to identify and reach at-risk children at both subnational and individual levels. Developing a robust algorithm to predict ZD children based on a set of variables could provide a valuable foundation for tailored interventions. Machine learning (ML) has emerged as a transformative tool in public health research particularly suited for this task which can capture complex relationships and interactions between variables [20-22]. Unlike traditional statistical methods that rely on predefined hypotheses, ML models can autonomously identify patterns and relationships within large datasets by learning from data rather than making prior assumptions [20,23,24]. This capability is particularly useful for multifactorial issues such as immunization uptake [25].

Using rule-based ML models can uncover hidden relationships among determinants of ZD children in large datasets, often represented through “if-then” statements that illustrate connections between variables [26]. This application of ML bridges the gap between theoretical research and practical applications, leading to advancements in the health care field [27].

This study aims to use ML algorithms to predict which Ethiopian children aged 12-35 months are at higher risk of being ZD and assess the predictive capabilities of the developed models. Findings from this study may provide actionable insights for policy makers and immunization program actors, informing the development of targeted strategies to effectively identify and reach those most at-risk children.

## Methods

### Study Design

The data for this study were sourced from the recent National Immunization Evaluation Survey in Ethiopia, which provides nationwide representation [28]. The survey included 11 regions and the 2 city administrations. A 2-stage stratified cluster sampling technique was used to select participants. The first stage is the enumeration areas (EAs), which served as clusters, randomly chosen with an urban-rural stratification approach, and the second stage is households within each EA. Sampling frames were prepared for each region and city administrations by the Ethiopian Statistical Services. The number of EAs required per region and city administration was determined based on the size within the stratum (study regions) and proportion of the Ethiopia population living in urban and rural areas (21.4% urban and 78.6% rural). A total of 468 EAs were randomly selected, comprising 100 from urban areas and 368 from rural regions, resulting in a total sample size of approximately 13,666 households with children aged 12‐35 months.

We extracted information on immunization status for children aged 12‐35 months. The vaccination status of children was assessed using 3 sources of information: caregiver reports, home-based vaccination cards, and facility-based records, following World Health Organization guidelines [29]. If a mother or a caregiver presented an immunization card, the child’s vaccination status was assessed from that card. In cases where the card was unavailable, data collectors were instructed to verify the information at the nearest health facility if the caregiver reported that their child had been vaccinated. The mother’s or caregiver’s self-reports were considered only when neither the immunization card nor the facility records were available.

Using the operational definition set by Gavi, we defined a variable ZD status for each child, which is set to 1 if the child did not receive the first dose of the diphtheria, TTs, and pertussis-containing vaccine (DPT1), and set 0 otherwise [30,31].

We included a set of predictor variables or features to capture characteristics that have been associated with ZD status (Table 1). The factors influencing the outcome of interest are grouped into 3 groups: socioeconomic and demographic variables, health service utilization, and perceptions and attitudes. The first group of socioeconomic and demographic variables encompasses individual, household, and community-level characteristics that may affect the outcome of interest. The health service utilization represents the access to and use of various health care services, which can impact immunization status. The third category focuses on the perceptions or attitudes that individuals or caregivers have toward the benefits of immunization. All the 3 categories of the variables gathered during the survey.

**Table 1.** The predictor variables used for analysis were extracted from the recent National Immunization Evaluation Survey in Ethiopia, 2023.

Category	Description	Response/type of data
Socioeconomic and demographic factor		
Residency	Type of living arrangement	Categorical (urban and rural)
Region	Geographic area of residence	Nominal (eg, Afar, Amhara...)
Religion	Cultural beliefs influencing health behaviors	Nominal or categorical (orthodox, Muslim, protestant, and others)
Marital status	Relationship status of the mother or the caregiver	Categorical (married and living together, married, married but not living together, and not in marital union)
Mother’s or caregiver’s educational status	Level of formal education attained	Categorical (no, primary, secondary, and higher education)
Occupation of mothers or caregivers	Employment status and type of work	Nominal categorical data
Birth order	Position of a child in relation to their siblings within a family	Categorical (first, second, third, and fourth and above)
Wealth index	Measures economic status	Categorical (poor, middle, and rich)
Health service utilization		
ANC^a^ follow-up	History of ANC visits for the index child	Categorical (Yes/No)
History of maternal tetanus diphtheria vaccine	Previous vaccinations received	Categorical (Yes/No)
Distance to immunization site	Perceived impact of distance on immunization access	Categorical (“big problem,” “not a problem”)
Place of delivery	Location where the child was born	Categorical (Home/Facility)
Postnatal care	Follow-up care received after childbirth	Categorical (Yes/No)
Perceptions and attitudes		
Mother’s or caregiver’s perceived benefits on immunization	Beliefs regarding the advantages of vaccination	Was Likert (categorized into poor or good)
Trust in health care provider	The belief of mothers or caregivers on the services provided	Categorized into poor or good

a ANC: antenatal care.

### Data Preprocessing and Transformation

**Figure 1.**
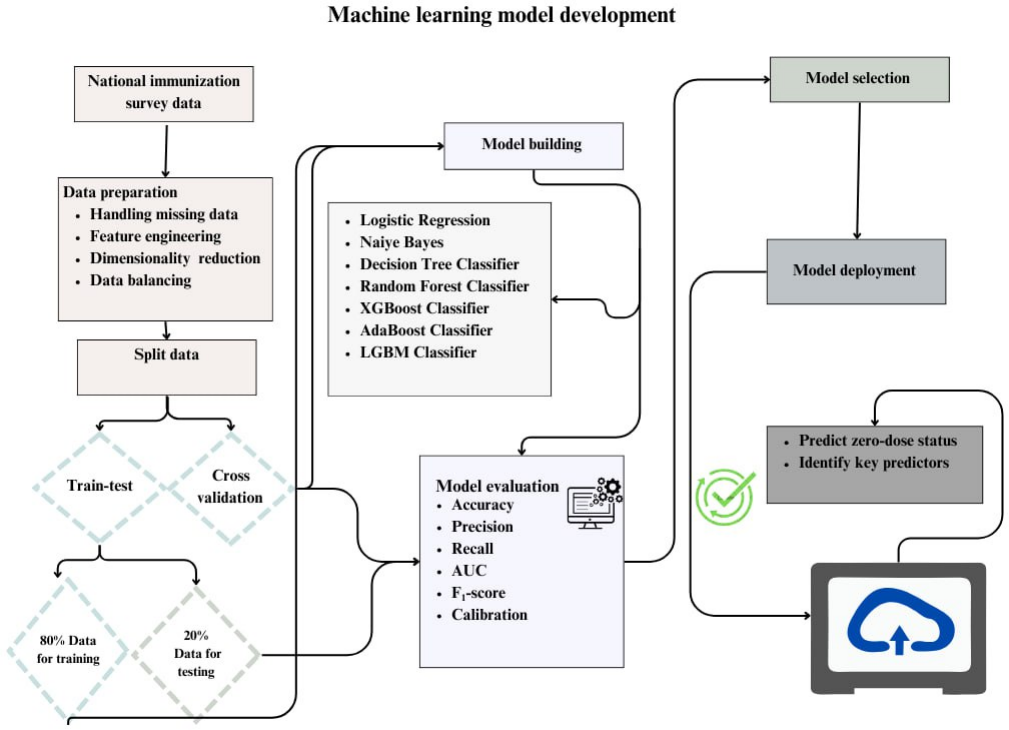
Data preparation and analysis steps for zero-dose children prediction. AUC: area under the curve; LGBM: Light Gradient Boosting Machine.

We implemented several preprocessing steps to enhance model performance. First, we addressed missing values in the independent variables using the k-nearest neighbor approach. We then transformed categorical variables into numerical format through one-hot encoding, which is essential for preparing data for ML models. To standardize feature ranges, we applied minimum-maximum scaling and mean normalization, ensuring comparability among features (Figure 1).

We conducted sampling weight as instance weights during the training process for all algorithms. This was done by using the sample_weight parameter in the model’s fitting functions, which adjusts the influence of each observation based on its probability of selection.

**Figure 2.**
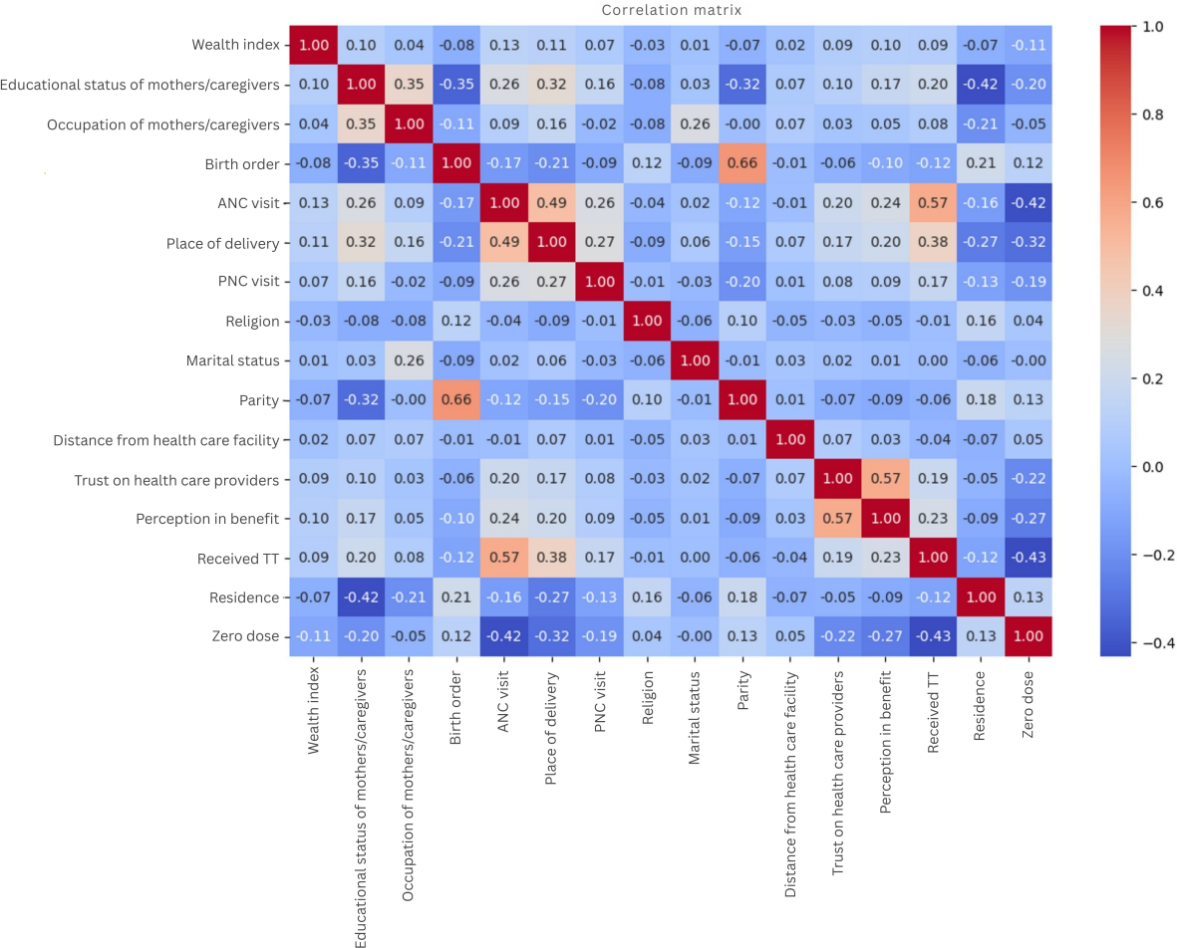
Correlation analysis matrix for predictor variables for zero-dose children, Ethiopia, 2023. ANC: antenatal care; PNC: postnatal care; TT: tetanus toxoid.

**Figure 3.**
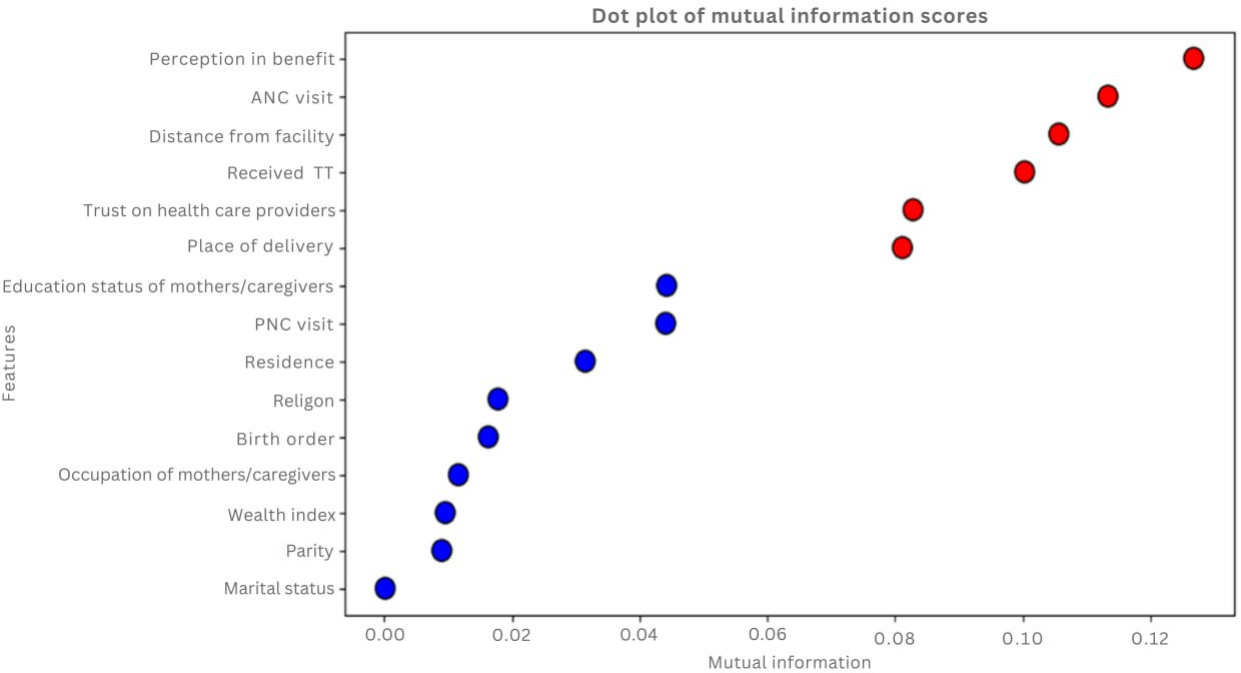
Mutual information score of predictor variables for zero-dose children, Ethiopia, 2023. ANC: antenatal care; PNC: postnatal care.

Next, we conducted a correlation analysis to identify and remove highly correlated features, thereby reducing multicollinearity and enhancing model robustness. Our correlation matrix showed a strong relation between parity and birth order (Figure 2), leading us to compute mutual information scores for each variable (Figure 3). This analysis highlighted ANC utilization and TT vaccination as significant predictors, while features such as marital status were excluded due to their minimal information value. Consequently, we retained birth order and omitted parity based on their scores.

For dimensionality reduction, we used Forward Selection, Backward Elimination, and Recursive Feature Elimination methods. We opted for Recursive Feature Elimination due to its effectiveness in identifying the most significant predictors while simplifying the dataset. To address class imbalance, we applied the Synthetic Minority Oversampling Technique, which balanced the dataset from an initial skew of 82% majority and 18% minority to an equal distribution. This balancing supports the development of robust predictive models and mitigates bias toward the majority class (Multimedia Appendix 1).

### Model Development

After the preprocessing, we split the dataset into 80% for training and 20% for testing (Figure 1). To avoid overfitting and underfitting, we applied 10-fold cross-validation, dividing the data into 10-folds and using one for validation while training on the others. The final performance is averaged across all folds.

The outcome variable, known as the class, is a binary variable indicating ZD status. A ZD status of 1 denotes a ZD child, while 0 indicates a non-ZD child. We applied supervised learning algorithms to develop a model from the training data to accurately predict this outcome in the test data.

Given the categorical nature of the outcome variable, we used 7 classical classification algorithms: AdaBoost Classifier [32], Logistic Regression [33], Naive Bayes, Random Forest (RF) [34], Light Gradient Boosting Machine (LGBM) [35], Extreme Gradient Boosting (XGBoost) [35], and Decision Tree [36]. These models generate a predicted score between 0 and 1 for each child, which is then classified as ZD or non-ZD based on a defined threshold. Following the initial model comparison, hyperparametric tuning was conducted to further optimize the performance of the best performing algorithm using a RandomizedSearchCV with cross-validation. The search involved 100 iterations with each hyperparameter combination evaluated using 5-fold cross-validation. Finally, the performance of each model was tested before and after balancing the dataset to choose the best predictive model. The model comparison was carried out using the balanced dataset.

### Model Evaluation

We evaluated model performance using both train-test split and cross-validation techniques, emphasizing both discrimination and calibration metrics to compare our classification of ZD status against the true ZD status of each child. Discrimination metrics included accuracy, precision, recall (sensitivity), *F*_1_-score, and area under the curve and area under the receiver operating characteristic curve. Accuracy reflects the proportion of correctly classified instances among all tested cases [37], while precision indicates the ratio of true-positive predictions to all positive predictions [38]. Recall measures the proportion of actual positive cases that the model successfully identifies [39], and the *F*_1_-score provides a balanced assessment of model performance, particularly useful in scenarios with class imbalances. In our application, as the objective is to assess the ability of a model to distinguish between positive and negative classes, area under the curve and area under the receiver operating characteristic curve emerged as the most critical measure, as it evaluates the model’s ability to effectively distinguish between positive and negative classes by analyzing the trade-off between sensitivity and specificity [40].

**Figure 4.**
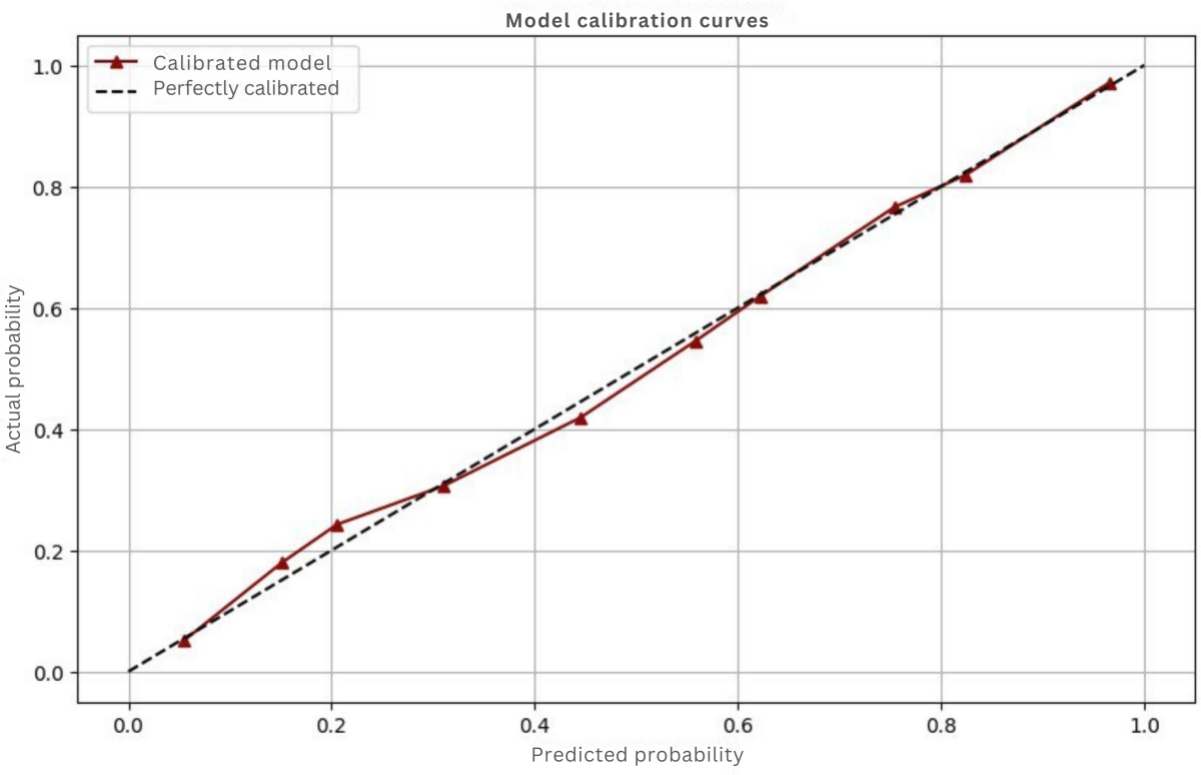
Calibration plot.

In addition to discrimination metrics, we performed calibration to examine how well the predicted probabilities align with actual outcomes. While a model can demonstrate good discrimination, it may still exhibit biases in its risk predictions [41]. Calibration is essential to ensure that predicted probabilities accurately reflect the likelihood of outcomes. To visualize this alignment, we used calibration curves, which plot predicted probabilities against observed results (Figure 4). An ideally calibrated model would form a 45-degree diagonal line, signifying that predicted probabilities correspond closely to actual outcomes [42].

### Important Feature Selection

Our second objective is to identify the most important predictors of ZD children. To achieve this, we used the best-performing ML model to determine the key features associated with identifying ZD cases. We used a unified framework developed by Lundberg and Lee [43], known as SHAP (SHapley Additive Explanations). This approach is based on Shapley values from cooperative game theory, which assign a value to each feature based on its contribution to the prediction, taking into account all possible combinations of features [44]. A waterfall plot is then created to visualize the cumulative effect of individual features on specific predictions, illustrating how each feature influences the final output. In addition, a beeswarm plot summarizes the distribution of SHAP values across multiple instances, revealing the variability and significance of feature contributions.

### Rule Generation

We used rule mining techniques to uncover patterns and relationships within our dataset. We used association rule mining to identify correlations between features through Apriori algorithms [45]. In addition, we applied classification rule mining to generate rules that predict class labels, aiding in the identification of key predictors for ZD children, and explored sequential rule mining to capture temporal patterns where relevant. Following the mining process, we generated actionable insights by formulating human-readable rules that outline conditions (antecedents) and outcomes (consequents) [46]. We assessed the quality of these rules using metrics such as confidence and lift to ensure their reliability and relevance [47].

### Ethical Considerations

The research was implemented in compliance with national and international ethical principles. The University of Gondar has provided ethical approval (CMHSSH-UOG IRERC/3/7/2024) to conduct this analysis. For this analyses we used the existing data with primary consent. We used deidentified data (summary data without individuals’ identity) to ensure confidentiality. We followed the international standard of strengthening the reporting of cross-sectional studies in epidemiology.

## Results

### Children’s and Mothers’ or Caregivers’ Characteristics

A total of 13,666 samples of children aged from 12 to 35 months were included for analysis. Nearly 57% (7727/13,666) of the children were younger than 24‐35 months. The majority (10,204/13,666, 74.7%) of the children were from mothers or caregivers who live in rural areas. Half (6986/13,666, 51.1%) of the children were born from mothers who had not had formal education. More than half (6757/12,419, 54.4%) of the children were from mothers who had no PNC follow-up for the index children. The details are shown in Table 2.

**Table 2.** Sociodemographic and economic characteristics of mothers or caregivers of children aged 12‐35 months in Ethiopia, 2023 (N=1366).

Variables	Frequency	Percentage
Age of the child		
12‐23 months	5934	43.5
24‐35 months	7727	56.5
Place of residency		
Rural	10,204	74.7
Urban	3462	25.3
Religion		
Orthodox	4430	32.4
Muslim	6158	45.2
Protestant	2944	21.5
Others^a^	134	1.0
Educational status		
No education	6986	51.1
Primary	3870	28.3
Secondary	1798	13.2
College and above	1012	7.4
Wealth status		
Poor	4558	33.4
Middle	4566	33.4
Richer	4542	33.2
Marital status		
Married and living together	12,765	93.4
Married but not living together	352	2.6
Not in marital union	549	4.0
Birth order		
First	4043	29.6
Second	4830	35.3
Third	2639	19.3
Fourth and above	2154	15.8
Parity		
Primipara	2499	20.1
Multipara (2-4)	6573	52.9
Grand multipara (5+)	3346	27.0
Perceived distance to health facility		
Big problem	5251	38.4
Not big problem	8415	61.6
Perceived benefit on immunization		
Poor	2502	19.3
Good	10,471	80.7
ANC^b^ visit		
Yes	10,345	83.3
No	2074	16.7
Place of delivery		
Home	3807	30.6
Health facility	8612	69.4
PNC^c^		
Yes	5662	45.6
No	6757	54.4

a Others: Catholic, traditional, and others.

b ANC: antenatal care.

c PNC: postnatal care.

### ZD Prevalence

**Figure 5.**
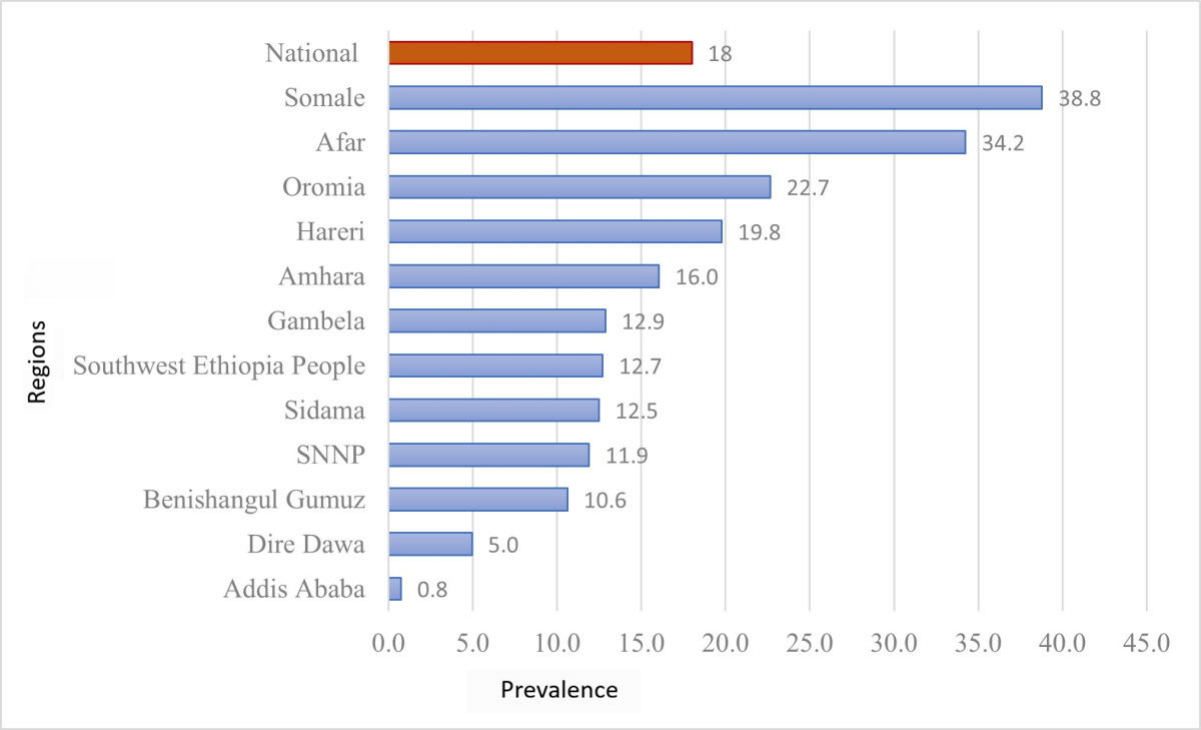
Distribution of zero-dose children aged 12‐35 months across regions in Ethiopia 2023. SNNP: Southern Nations, Nationalities, and Peoples'..

The overall prevalence of ZD in Ethiopia was 18% (95% CI 17.4%‐18.7%). There were regional variations in the prevalence of ZD children. The higher prevalence was observed in Somali (38.8%), Afar (34.2%), and followed by Oromia (22.7%), and the lowest observed in Addis Ababa (0.8%) and Dire Dawa (5 %) (Figure 5).

### Performance of the Prediction Models

Seven ML algorithms were used to predict ZD status in Ethiopia, with the LGBM yielding the best performance for both unbalanced and balanced datasets (Table 3). It achieved accuracies of 89% and 93% for the unbalanced and balanced datasets, respectively. Most models showed improved accuracy when applied to the balanced dataset, except for Logistic Regression and Naive Bayes. After balancing the data, both XGBoost and LGBM reached an accuracy of 93%. Notably, the LGBM classifier excelled in terms of area under the curve (AUC) (98%) and sensitivity (92%).

**Table 3.** Model performance comparison before and after dataset balancing for predicting zero-dose children in Ethiopia, 2023.

Models and dataset	Accuracy (%)	AUC^a^ (%)	Precision (%)	Sensitivity	*F*_1_-score
Logistic Regression					
Unbalanced	88	88	77	48	59
Balanced	81	89	83	77	80
Naïve Bayes					
Unbalanced	85	87	59	63	61
Balanced	79	87	82	73	77
LGBM^b^ Classifier					
Unbalanced	89	88	79	53	63
Balanced	93	97	94	91	92
DT^c^ Classifier					
Unbalanced	86	75	64	51	57
Balanced	89	91	90	87	88
Random Forest Classifier					
Unbalanced	87	85	70	52	61
Balanced	91	96	91	90	91
XGBoost Classifier					
Unbalanced	88	87	75	52	61
Balanced	93	97	94	90	92
AdaBoost Classifier					
Unbalanced	88	88	77	46	58
Balanced	88	95	89	86	87

a AUC: area under the curve.

b LGBM: Light Gradient Boosting Machine.

c DT: Decision Tree.

Overall, while all ML models performed well on both datasets, those trained on balanced data especially XGBoost and LGBM proved to be more effective in identifying ZD children due to their higher recall and AUC. A comprehensive comparison of the ML algorithms used for ZD children is detailed in Table 3. After the hyperparameter optimization conducted, the LGBM model achieved robust performance, with an accuracy of 92.4, an AUC of 97.4%, a precision of 93.2%, and a recall of 90.9%. The details are shown in Table 4.

**Table 4.** Model performance after hyperparameter tuning for predicting zero-dose children in Ethiopia, 2023.

Model	Accuracy (%)	AUC^a^ (%)	Precision (%)	Recall (%)	*F*_1_-score (%)
Logistic Regression	80.8	89.1	82.5	76.9	79.6
Naive Bayes	79.1	87.3	82	73.1	77.3
Random Forest	91.6	96.7	91.9	90.6	91.3
XGBoost	92.2	97.3	93.9	89.8	91.8
AdaBoost	89.6	96.2	90.6	87.8	89.2
LGBM^b^	92.4	97.4	93.2	90.9	92.1
Decision Tree	89.9	94.5	89.5	89.7	89.6

a AUC: area under the curve.

b LGBM: Light Gradient Boosting Machine.

**Figure 6.**
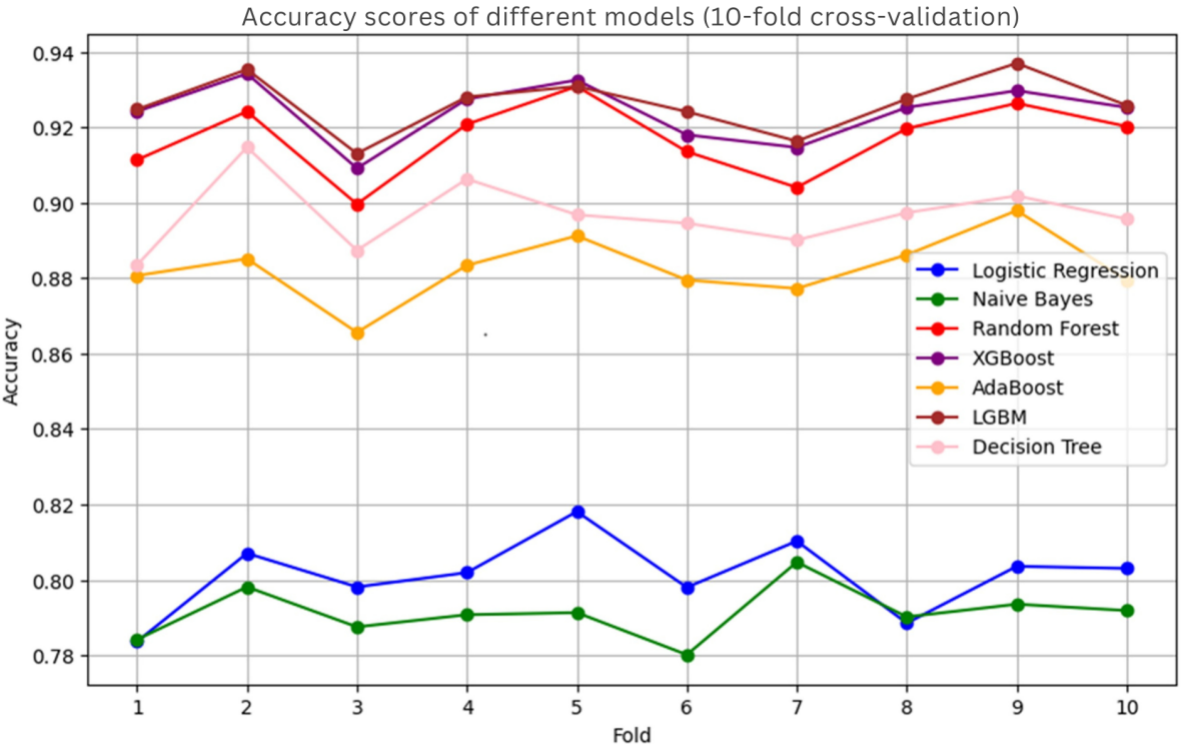
Accuracy of models in 10-fold cross-validation after balancing the dataset for predicting zero-dose children in Ethiopia, 2023. LGBM: Light Gradient Boosting Machine.

After parameter tuning, the models were further evaluated using 10-fold cross-validation, where XGBoost and LGBM demonstrated comparable accuracies of 93% (Figure 6).

### Predicting ZD

After building the model by using the training dataset, the performance of the LGBM model was evaluated by the testing dataset. From 2181 ZD children, the model predicted 1991 children correctly (true positive), and out of 2300 non-ZD children, the model predicted 2175 children correctly (true negative). However, the model incorrectly classified 190 ZD samples as non-ZD (false positive) and 125 non-ZD samples as ZD (false negative). The Matthews correlation coefficient was *r*=0.85 and Cohen κ=0.85. Overall, the model predicted with an accuracy of 93%, recall of 91%, *F*_1_-score of 92%, and 94% precision on test data.

### Feature Importance

**Figure 7.**
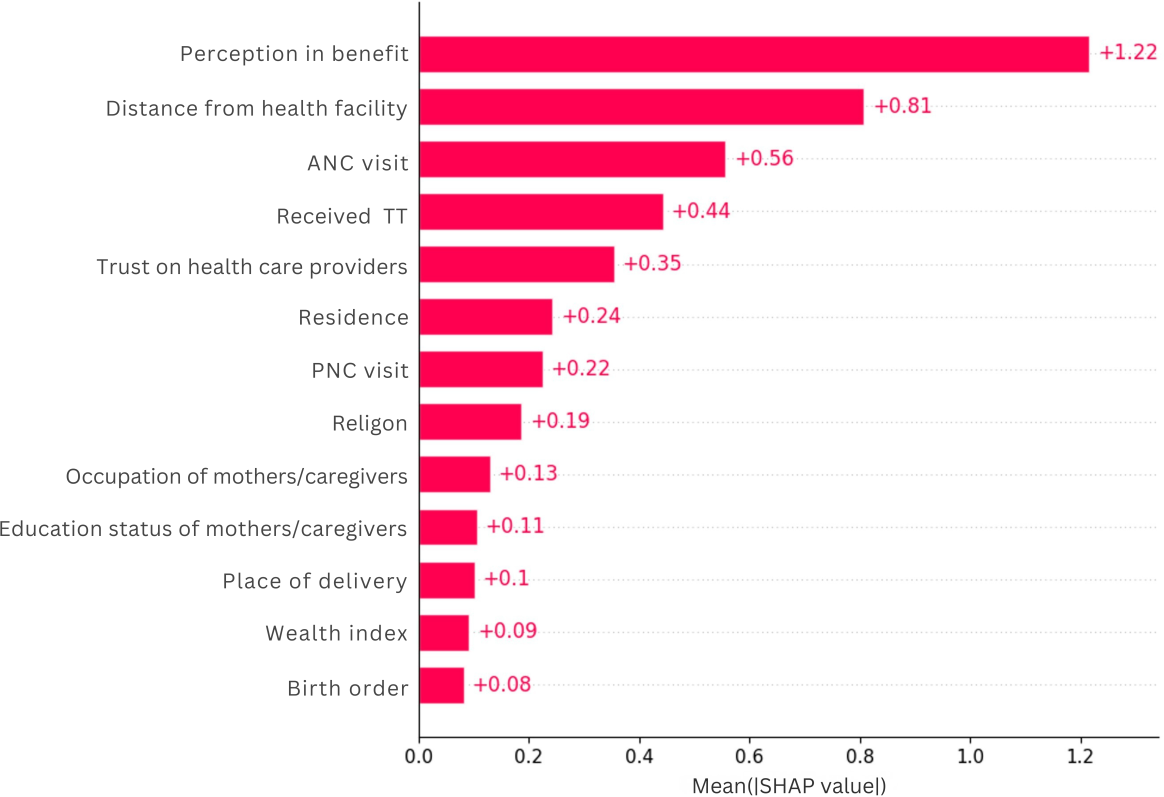
Important features for predicting zero-dose children in Ethiopia, 2023. ANC: antenatal care; PNC: postnatal care; SHAP: SHapley Additive Explanations.

The Shapley Additive analysis identified that mother’s or caregiver’s perception of benefit of immunization (+1.13), with whether the distance to immunization site (+0.88), whether the mother received ANC (+0.55), whether the mother received TT (+0.42), and whether trust in health providers (0.41) were the most important features followed by place of residence (+0.35), and PNC visit (+0.25). Wealth index, birth order, and place of delivery were the features with low importance (Figure 7).

**Figure 8.**
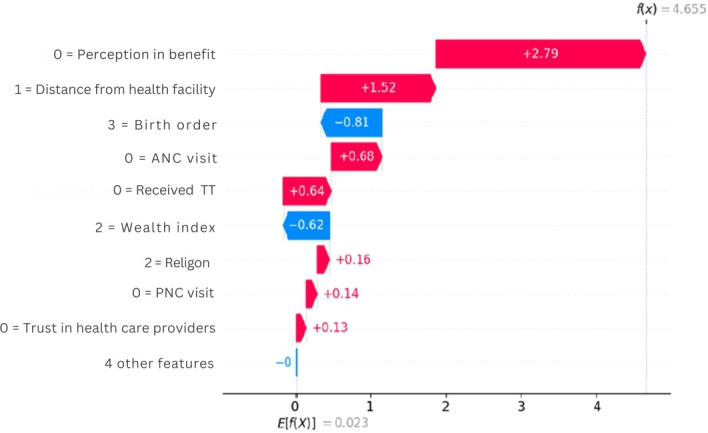
Waterfall plot of first observation value to predict zero-dose children in Ethiopia, 2023. ANC: antenatal care; PNC: postnatal care.

The waterfall chart demonstrates how various factors influence the prediction of ZD vaccination status, starting from a baseline expected value of (*E*[*f*(*X*)]=0.023) and culminating in a final prediction of *f*(*x*)=4.655) indicating that the child is ZD. This indicated that poor perceptions of vaccination benefits, long distances to immunization sites, lack of antenatal and postnatal care visits, absence of TT vaccination, and low trust in health care providers are positively correlated with ZD. Conversely, being in a medium wealth index and having a third birth order is negatively correlated with ZD (Figure 8).

.

**Figure 9.**
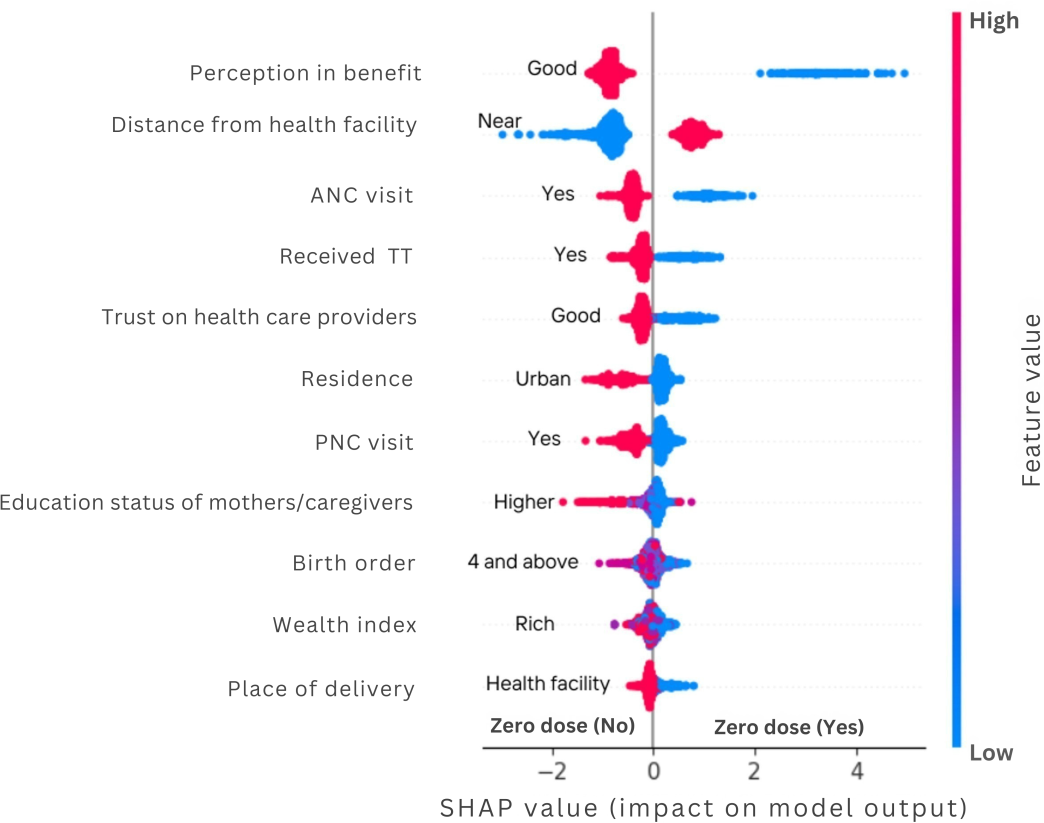
Zero-dose predictors for Light Gradient Boosting Machine model. SHAP summary plot of top predictors. ANC: antenatal care; PNC: postnatal care; SHAP: SHapley Additive Explanations.

As shown in Figure 9, the beeswarm plot illustrates the impact of various predictor variables on ZD status, with distinct colors representing risk levels: red dots indicate high-risk values, while blue dots denote low-risk values for the predictor variables. The feature of perception exhibits a wide range of SHAP values, highlighting its significant influence on the model’s predictions. A poor perception of the benefits of vaccination notably increases the likelihood of a child being classified as ZD. In addition, distance from health care facilities is strongly associated with ZD status, where far distances correlate with a higher likelihood of being unvaccinated. Other contributing factors include a lack of ANC visits, PNC visits, TT vaccination, low wealth index, low trust in health care providers, and home delivery, all of which contribute to the prediction of the positive class (ZD).

### Rule Generation

The rule generation process was done based on important attributes selected by the best performing ML model LGBM. Moving beyond individual feature importance, we used association rule mining to identify complex, multifactorial profiles of ZD children and to rigorously validate the interactions suggested by the SHAP analysis. This generated a set of human-interpretable “if then” rules, each validated by key metrics: support (prevalence of the rule in the data), confidence (conditional probability of the outcome), and lift (strength of the association above random chance). The rule generation process was done based on important attributes selected by the best performing ML model LGBM. The strongest rule (rule 1: lift = 2.17, confidence = 0.90) indicates that children whose caregivers live far from a health facility and have a poor perception of vaccination benefits have a 90% probability of being ZD, a risk 2.17 times higher than random. Rule 2 (confidence = 0.81) shows that combining distance with a lack of ANC and poor trust in providers creates another high-risk pathway, while rule 5 (confidence = 0.79) highlights the potent combination of no tetanus vaccination, no ANC, and distance. Crucially, these rules reveal critical synergies, while SHAP identified “distance” and “ANC” as top individual predictors, rule mining quantified how their combination with other factors (eg, rule 8: no ANC + Far distance, confidence = 0.72) creates a risk profile with a distinctly high probability of the outcome. This provides programmatically actionable insights, demonstrating that interventions must target these intersecting barriers simultaneously rather than in isolation to effectively reach ZD children. A total of 9 association rules were generated, and the details of the rules are shown in Textbox 1.

Textbox 1. **Rule generation and knowledge extraction.**

##Rule## 1: Distance from facility_far, Perception in benefit_Poor -> Zerodose_Yes

Support: 0.10897435897435898, Lift: 2.1738917080243128, Confidence: 0.9037974683544304

##Rule## 2: Distance from facility_far, Trust in healthcare provider_Poor, Anc visit_No -> Zerodose_Yes

Support: 0.10134310134310134, Lift: 1.947695283120232, Confidence: 0.8097560975609757

##Rule## 3: Perception in benefit_Poor -> Zerodose_Yes

Support: 0.15262515262515264, Lift: 1.9211552265274243, Confidence: 0.7987220447284347

##Rule## 4: Perception in benefit_Poor, Place Residence_rural -> Zerodose_Yes

Support: 0.10103785103785104, Lift: 1.9138215859030838, Confidence: 0.795673076923077

##Rule## 5: Received TT_No, Distance from facility_far, Anc visit_No -> Zerodose_Yes

Support: 0.13064713064713065, Lift: 1.9099490817552491, Confidence: 0.7940630797773656

##Rule## 6: Received TT_No, Distance from facility_far, Place delivery_Home -> Zerodose_Yes

Support: 0.12606837606837606, Lift: 1.7738986784140967, Confidence: 0.7374999999999999

##Rule## 7: PNC visit_Yes, Distance from facility_far, Anc visit_No -> Zerodose_Yes

Support: 0.10073260073260074, Lift: 1.7406677486668212, Confidence: 0.7236842105263158

##Rule## 8: Distance from facility_far, Anc visit_No -> Zerodose_Yes

Support: 0.17918192918192918, Lift: 1.736658159533137, Confidence: 0.7220172201722017

##Rule## 9: Distance from facility_far, Anc visit_No, Place Residence_rural -> Zerodose_Yes

Support: 0.1108058608058608, Lift: 1.7019862431408919, Confidence: 0.7076023391812866

## Discussion

### Principal Findings

Using the data from the most recent National Immunization Evaluation Survey in Ethiopia, we applied different supervised machine algorithms to assess how well the models predict whether a child is likely to be ZD and to identify the important predictor variables. We trained and compared 7 ML classifiers on both unbalanced and balanced datasets, using a train-test split, hyperparameter tuning, and 10-fold cross-validation for robust evaluation. A variety of socioeconomic, demographic, and health-related factors were included to enhance the model’s predictions and facilitate important feature selection.

Our findings demonstrate that these ML algorithms are effective in identifying children at high risk of being ZD. Among the 7 models tested, LGBM emerged as the top performer, achieving an AUC of 97.4%, recall of 90.9%, accuracy of 92.4%, precision of 93.2%, and an *F*_1_-score of 92.1%. These evaluation metrics underscore the model’s strong capability in predicting ZD children. The high AUC indicates the model’s effectiveness in distinguishing between children who receive immunization services and those who do not. Notably, a recall of 90.9% signifies that the model successfully identifies 90.9% of ZD children, who are often at greater risk for missing vaccines and vaccine-preventable diseases.

In addition to LGBM, both XGBoost and RF algorithms performed well, each achieving an accuracy of 92.2% and 91.6%, respectively. These results are consistent with previous studies that recognized XGBoost [48] and RF [49] as top performers in similar contexts. While these metrics indicate robust performance for critical health issues such as immunization, it is crucial to validate the model in real-world settings. Such testing will enhance its utility as a tool for guiding public health initiatives aimed at increasing vaccination rates and improving access to essential health care services for unvaccinated children.

Using an ML model, health care workers can pinpoint specific households and communities with ZD children, allowing them to shift from broad campaigns to targeted household visits. By leveraging the model’s insights on local perceptions and socioeconomic barriers, they can tailor their communication and services, such as setting up mobile clinics, to overcome specific challenges and efficiently use scarce resources, ensuring that vaccines reach those most in need.

The second objective of the study was to identify important attributes that could predict ZD among children aged 12‐35 months. Using SHAP analysis, the study found that perception of immunization benefit, ANC utilization, distance from vaccination site, maternal TT vaccination status, and trust in health providers were the most important features to identify at-risk children for ZD.

The top predictor was poor maternal perception with a SHAP value of 1.13 (Figure 7). This indicates that a negative perception of mothers or caregivers increases the likelihood of a child being ZD, likely because parental beliefs directly influence health care decisions regarding vaccination. This finding aligns with previous studies showing that parental beliefs and attitudes significantly affect a child’s vaccination status [50,51].

ANC utilization was another important feature, with a SHAP value of 0.55, indicating that a lack of ANC is strongly linked to a child being ZD. This finding is in line with the previous similar studies done [48,52,53]. This could be due to the fact that ANC visits enable mothers to access integrated health services, be more likely to receive information on immunization schedules, build trust in the health system, and improve adherence to health services [54-56].

In addition, the study found several important predictors. Maternal TT vaccination was a key factor; mothers who received the TT vaccine were more likely to have their children vaccinated, a finding consistent with studies from Sudan and Bangladesh [57,58]. Postnatal care visit was another important predictor. This service is likely gaining a better understanding of vaccination importance and feeding practices, thus reducing missed vaccinations [59,60]. In addition, maternal education was an important predictor, with uneducated mothers having a higher risk of ZD children than those with at least a primary education, firming up the known link between maternal literacy and vaccination rates and primary education. This finding is in line with previous research linking maternal literacy to vaccination completeness [61-65]. The other finding of this study is rule mining and generation. Using association rule mining with the Apriori algorithm, the study uncovered strong relationships between various socioeconomic, demographic, and health-related factors and ZD status. Key determinants, including distance from health facility, perception of vaccination benefits, trust in health care providers, ANC, place of delivery, place of residency, and TT vaccines were the most important features predicting ZD. Confidence levels for these findings ranged from 71% to 90%, indicating robust associations.

Findings from association rule 1 indicated that the probability of a child being ZD would be 90%, if and only if the mothers or caregivers were far from the health facility and had poor perception on immunization. This may be because mothers or caregivers who are far from the facility may not have access to health education directly or indirectly, affecting health-seeking behavior and health service utilization such as vaccination. The second rule also included poor trust in health care providers and lack of ANC visits as predictors for ZD. A child ZD would be 80% if mothers or caregivers have trust in providers and had no ANC follow-up for the index child.

### Strengths and Limitations

This study had several strengths worth mentioning. We used national-level survey data from 463 EAs ensuring generalizability across the country and providing a current snapshot of the ZD situation. A key strength is that our analysis uses various ML algorithms from the field of data science, which significantly aids in identifying and targeting ZD children more effectively. These advanced analytical techniques allow us to process large datasets and uncover insights that may not be immediately apparent through traditional methods. At the same time, this study identified the risk factors of ZD that may help policy makers and planners to design tailored interventions to identify and reach the unvaccinated children.

This study was subject to some limitations. First, although we used national-level data, we did not include data from the Tigray region, which is one of the administrative regions of the country, due to security issues. Second, the study did not include health system side predictors such as availability of vaccination supplies and vaccines. Finally, we could not do external validation for the modes due to the lack of real-world data.

### Conclusions

The developed ML models effectively predict children at risk of being ZD and identify associated risk factors. Among these models, the LGBM model demonstrated the best performance in predicting ZD children. Key features linked to ZD status include access to immunization sites, maternal health service utilization (such as antenatal and postnatal care, place of delivery, and TT vaccination), and perceptions regarding immunization.

By implementing ML models, public health interventions can be more precisely targeted at the most vulnerable groups. This approach may address inequities in vaccination coverage by identifying specific sociodemographic, economic, and health-related factors associated with ZD children. Consequently, it aids in the formulation and implementation of effective policies and strategies to improve vaccination rates. Strengthening the continuum of care for mothers, raising awareness among caregivers, and improving immunization access through outreach strategies may help in reducing the high burden of ZD children.

## Supplementary material

10.2196/76712 Multimedia Appendix 1 Zero-dose status among children aged 12‐35 months in Ethiopia, before and after data balancing, using the 2023 survey dataset. SMOTE: Synthetic Minority Oversampling Technique.

## Abbreviations

ANC: antenatal care
AUC: area under the curve
DPT: diphtheria, tetanus, and pertussis
EA: enumeration area
LGBM: Light Gradient Boosting Machine
ML: machine learning
RF: Random Forest
SHAP: SHapley Additive Explanations
TT: tetanus toxoid
XGBoost: Extreme Gradient Boosting

## References

[1] Clark H, Coll-Seck AM, Banerjee A (2020). A future for the world’s children? A WHO–UNICEF–Lancet Commission. Lancet.

[2] Kaur G, Danovaro-Holliday MC, Mwinnyaa G (2023). Routine vaccination coverage—worldwide, 2022. MMWR Morb Mortal Wkly Rep.

[3] Sharrow D, Hug L, You D (2022). Global, regional, and national trends in under-5 mortality between 1990 and 2019 with scenario-based projections until 2030: a systematic analysis by the UN Inter-agency Group for Child Mortality Estimation. Lancet Glob Health.

[4] (2023). WHO/UNICEF estimates of national immunization coverage. World Health Organization.

[5] (2020). Immunization Agenda 2030: a global strategy to leave no one behind. https://www.who.int/publications/m/item/immunization-agenda-2030-a-global-strategy-to-leave-no-one-behind.

[6] Restrepo-Méndez MC, Barros AJ, Wong KL (2016). Inequalities in full immunization coverage: trends in low- and middle-income countries. Bull World Health Organ.

[7] Ozawa S, Yemeke TT, Evans DR, Pallas SE, Wallace AS, Lee BY (2019). Defining hard-to-reach populations for vaccination. Vaccine (Auckl).

[8] Johri M, Rajpal S, Subramanian SV (2021). Progress in reaching unvaccinated (zero-dose) children in India, 1992-2016: a multilevel, geospatial analysis of repeated cross-sectional surveys. Lancet Glob Health.

[9] Johri M, Ng ES, Sharkey A, Bosson-Rieutort D, Kone GK, Subramanian SV (2023). Effects of zero-dose vaccination status in early childhood and level of community socioeconomic development on learning attainment in preadolescence in India: a population-based cohort study. bmjph.

[10] Chopra M, Bhutta Z, Chang Blanc D (2020). Addressing the persistent inequities in immunization coverage. Bull World Health Organ.

[11] Teferi E (2016). Factors influencing coverage and key challenges to achieving targets of routine immunization in Africa: a systematic review. Ethiop J Pediatr Child Health.

[12] Nour TY, Farah AM, Ali OM, Osman MO, Aden MA, Abate KH (2020). Predictors of immunization coverage among 12–23 month old children in Ethiopia: systematic review and meta-analysis. BMC Public Health.

[13] Tamir TT, Zegeye AF, Mekonen EG (2024). Prevalence, spatial variation and determinants of zero-dose children in Ethiopia: spatial and multilevel analyses. Public Health (Fairfax).

[14] Yadita ZS, Ayehubizu LM (2021). Full immunization coverage and associated factors among children aged 12-23 months in Somali Region, Eastern Ethiopia. PLoS One.

[15] Biset G, Woday A, Mihret S, Tsihay M (2021). Full immunization coverage and associated factors among children age 12-23 months in Ethiopia: systematic review and meta-analysis of observational studies. Hum Vaccin Immunother.

[16] Girmay A, Dadi AF (2019). Full immunization coverage and associated factors among children aged 12-23 months in a hard-to-reach areas of Ethiopia. Int J Pediatr.

[17] Gurmu E, Etana D (2016). Factors influencing children’s full immunization in Ethiopia. Afr Popul Stud.

[18] Asresie MB, Dagnew GW, Bekele YA (2023). Changes in immunization coverage and contributing factors among children aged 12-23 months from 2000 to 2019, Ethiopia: multivariate decomposition analysis. PLoS One.

[19] Nozaki I, Hachiya M, Kitamura T (2019). Factors influencing basic vaccination coverage in Myanmar: secondary analysis of 2015 Myanmar demographic and health survey data. BMC Public Health.

[20] Song X, Mitnitski A, Cox J, Rockwood K (2004). Comparison of machine learning techniques with classical statistical models in predicting health outcomes. Stud Health Technol Inform.

[21] Bonaccorso G (2018). Machine Learning Algorithms: Popular Algorithms for Data Science and Machine Learning.

[22] Sarker IH (2021). Machine learning: algorithms, real-world applications and research directions. SN Comput Sci.

[23] Agrawal R, Imieliński T, Swami A Mining association rules between sets of items in large databases.

[24] Cheong Q, Au-Yeung M, Quon S, Concepcion K, Kong JD (2021). Predictive modeling of vaccination uptake in US counties: a machine learning-based approach. J Med Internet Res.

[25] Carrieri V, Lagravinese R, Resce G (2021). Predicting vaccine hesitancy from area-level indicators: a machine learning approach. Health Econ.

[26] Avirappattu G, Pach Iii A, Locklear CE, Briggs AQ (2022). An optimized machine learning model for identifying socio-economic, demographic and health-related variables associated with low vaccination levels that vary across ZIP codes in California. Prev Med Rep.

[27] Zhang A, Xing L, Zou J, Wu JC (2022). Shifting machine learning for healthcare from development to deployment and from models to data. Nat Biomed Eng.

[28] (2024). National immunization program evaluation research in Ethiopia. https://www.scribd.com/document/875138308.

[29] (2018). World Health Organization vaccination coverage cluster surveys: reference manual. World Health Organization.

[30] Wonodi C, Farrenkopf BA (2023). Defining the zero dose child: a comparative analysis of two approaches and their impact on assessing the zero dose burden and vulnerability profiles across 82 low- and middle-income countries. Vaccines (Basel).

[31] Zero dose children and missed communities. Gavi.

[32] Schapire RE, Freund Y (2013). Boosting: foundations and algorithms. Kybernetes.

[33] Cessie SL, Houwelingen JCV (1992). Ridge estimators in logistic regression. Appl Stat.

[34] Pal M (2005). Random forest classifier for remote sensing classification. Int J Remote Sens.

[35] Chen T, Guestrin C XGBoost: a scalable tree boosting system.

[36] Breiman L, Friedman J, Olshen RA, Stone CJ (2017). Classification and Regression Trees.

[37] Šimundić AM (2009). Measures of Diagnostic Accuracy: Basic Definitions. EJIFCC.

[38] Flach P, Kull M (2015). Precision-recall-gain curves: PR analysis done right. Adv Neural Inf Process Syst.

[39] Santini A, Man A, Voidăzan S (2021). Accuracy of diagnostic tests. J Crit Care Med.

[40] Kumar R, Indrayan A (2011). Receiver operating characteristic (ROC) curve for medical researchers. Indian Pediatr.

[41] Collins GS, Reitsma JB, Altman DG, Moons KGM, TRIPOD Group (2015). Transparent reporting of a multivariable prediction model for individual prognosis or diagnosis (TRIPOD): the TRIPOD statement. The TRIPOD Group. Circulation.

[42] Huang Y, Li W, Macheret F, Gabriel RA, Ohno-Machado L (2020). A tutorial on calibration measurements and calibration models for clinical prediction models. J Am Med Inform Assoc.

[43] Lundberg SM, Lee SI (2017). A unified approach to interpreting model predictions. https://proceedings.neurips.cc/paper/2017/hash/8a20a8621978632d76c43dfd28b67767-Abstract.html.

[44] Bifarin OO (2023). Interpretable machine learning with tree-based SHapley Additive Explanations: application to metabolomics datasets for binary classification. PLoS One.

[45] Altaf W, Shahbaz M, Guergachi A (2017). Applications of association rule mining in health informatics: a survey. Artif Intell Rev.

[46] Khare S, Gupta D Association rule analysis in cardiovascular disease.

[47] Agrawal R, Srikant R (1994). Fast algorithms for mining association rules. https://www.columbia.edu/~rd2537/docu/apriori(abstract).pdf.

[48] Tadese ZB, Nigatu AM, Yehuala TZ, Sebastian Y (2024). Prediction of incomplete immunization among under-five children in East Africa from recent demographic and health surveys: a machine learning approach. Sci Rep.

[49] Chandir S, Siddiqi DA, Hussain OA (2018). Using predictive analytics to identify children at high risk of defaulting from a routine immunization program: feasibility study. JMIR Public Health Surveill.

[50] Chambongo PE, Nguku P, Wasswa P, Semali I (2016). Community vaccine perceptions and its role on vaccination uptake among children aged 12-23 months in the Ileje District, Tanzania: a cross section study. Pan Afr Med J.

[51] Puri YE, Murti B, Demartoto A (2016). Analysis of the effect of maternal perception on completeness of child immunization status with health belief model. J Health Promot Behav.

[52] Demsash AW, Chereka AA, Walle AD, Kassie SY, Bekele F, Bekana T (2023). Machine learning algorithms’ application to predict childhood vaccination among children aged 12–23 months in Ethiopia: evidence 2016 Ethiopian Demographic and Health Survey dataset. PLoS One.

[53] Biswas A, Tucker J, Bauhoff S (2023). Performance of predictive algorithms in estimating the risk of being a zero-dose child in India, Mali and Nigeria. BMJ Glob Health.

[54] Dixit P, Dwivedi LK, Ram F (2013). Strategies to improve child immunization via antenatal care visits in India: a propensity score matching analysis. PLoS One.

[55] Jiao B, Iversen I, Sato R (2024). Association between achieving adequate antenatal care and health-seeking behaviors: a study of demographic and health surveys in 47 low- and middle-income countries. PLoS Med.

[56] Krishnamoorthy Y, Rehman T (2022). Impact of antenatal care visits on childhood immunization: a propensity score-matched analysis using nationally representative survey. Fam Pract.

[57] Ibrahim ZA, Sabahelzain MM, Elhadi YAM, Malande OO, Babiker S (2023). Predictors of tetanus vaccine uptake among pregnant women in Khartoum State, Sudan: a hospital-based cross-sectional study. Vaccines (Basel).

[58] Amin MB, Roy N, Meem AE, Hossain E, Aktarujjaman M (2022). Trends and determinants of taking tetanus toxoid vaccine among women during last pregnancy in Bangladesh: country representative survey from 2006 to 2019. PLoS One.

[59] Laryea DO, Abbeyquaye Parbie E, Frimpong E (2014). Timeliness of childhood vaccine uptake among children attending a tertiary health service facility-based immunisation clinic in Ghana. BMC Public Health.

[60] Sarker AR, Akram R, Ali N, Chowdhury ZI, Sultana M (2019). Coverage and determinants of full immunization: vaccination coverage among Senegalese children. Medicina.

[61] Acharya P, Kismul H, Mapatano MA, Hatløy A (2018). Individual- and community-level determinants of child immunization in the Democratic Republic of Congo: a multilevel analysis. PLoS One.

[62] Rauniyar SK, Munkhbat E, Ueda P, Yoneoka D, Shibuya K, Nomura S (2020). Timeliness of routine vaccination among children and determinants associated with age-appropriate vaccination in Mongolia. Heliyon.

[63] Budu E, Darteh EKM, Ahinkorah BO, Seidu AA, Dickson KS (2020). Trend and determinants of complete vaccination coverage among children aged 12-23 months in Ghana: analysis of data from the 1998 to 2014 Ghana Demographic and Health Surveys. PLoS One.

[64] Noh JW, Kim Y m., Akram N (2018). Factors affecting complete and timely childhood immunization coverage in Sindh, Pakistan; a secondary analysis of cross-sectional survey data. PLoS One.

[65] Sarker AR, Akram R, Ali N, Sultana M (2019). Coverage and factors associated with full immunisation among children aged 12–59 months in Bangladesh: insights from the nationwide cross-sectional demographic and health survey. BMJ Open.

