# Supplementary figures and images for: Modeling Zero-Dose Children in Ethiopia: A Machine Learning Perspective on Model Performance and Predictor Variables
Source: JMIR Pediatr Parent. 2026 Feb 2;9:e76712. doi: 10.2196/76712 (PMC12863459; doi:10.2196/76712)

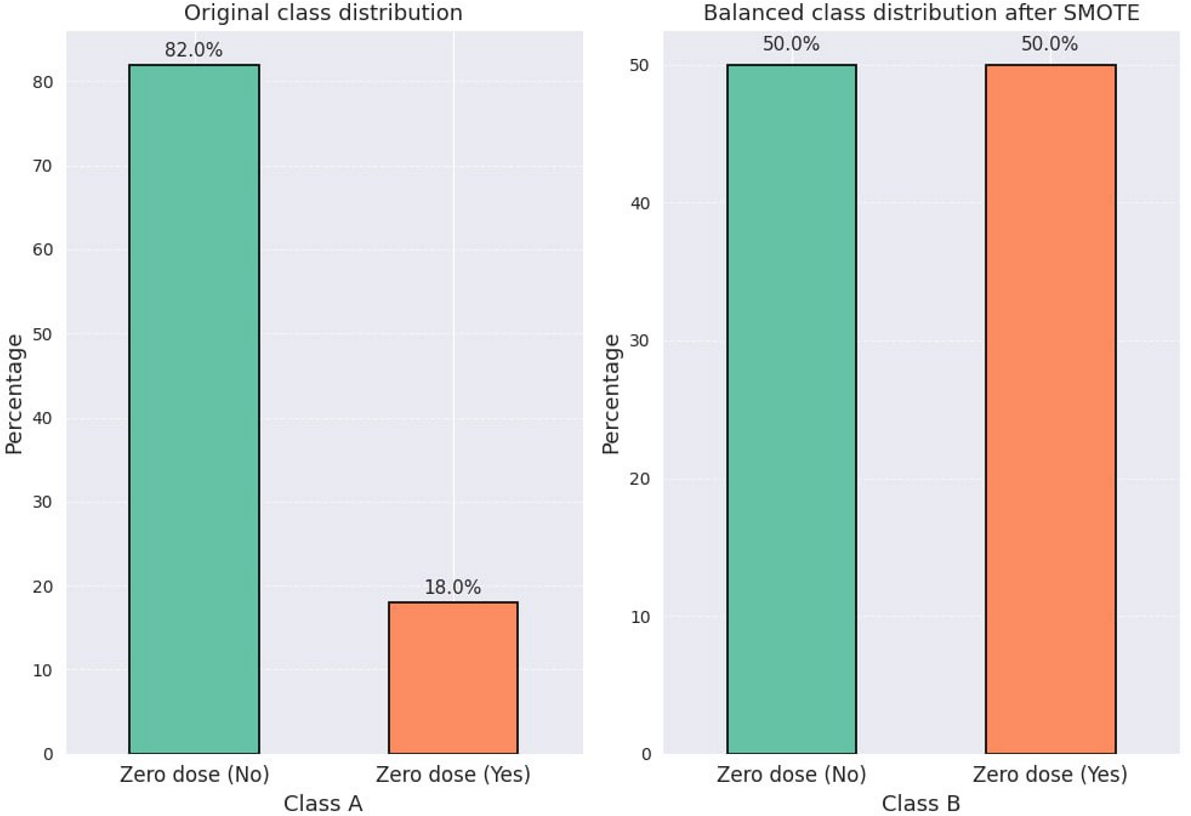

Supplement: Multimedia Appendix 1 [file pediatrics-v9-e76712-s001.png]
